# Characterization of Small, Mononuclear Blood Cells from Salmon Having High Phagocytic Capacity and Ability to Differentiate into Dendritic like Cells

**DOI:** 10.1371/journal.pone.0049260

**Published:** 2012-11-14

**Authors:** Gyri T. Haugland, Ann-Elise O. Jordal, Heidrun I. Wergeland

**Affiliations:** Department of Biology, University of Bergen, Bergen High-Technology Centre, Bergen, Norway; University of Iowa Carver College of Medicine, United States of America

## Abstract

Phagocytes are the principal component of the innate immune system, playing a key role in the clearance of foreign particles that include potential pathogens. In vertebrates, both neutrophils and mononuclear cells like monocytes, macrophages and dendritic cells are all professional phagocytes. In teleosts, B-lymphocytes also have potent phagocytic ability. We have isolated a population of small (<5 µm), mononuclear blood cells from Atlantic salmon (*Salmo salar* L.) not previously characterized. In order to identify them, we have performed morphological, gene expression, flow cytometry, cytochemical, ultrastructural and functional analyses. Interestingly, they highly express the gene encoding CD83, the most characteristic cell surface marker for dendritic cells in mammals, and MHC class II limited to professional antigen presenting cells. They did not express genes nor did they have cell markers for B-cells, T-cells, monocytes/macrophages or neutrophils as shown by qRT-PCR, flow cytometry and immunoblotting. A remarkable feature of these cells is their potent phagocytic capacity. Their oxygen-independent killing mechanism, as shown by intense acid phosphatase staining, is supported by lack of respiratory burst and myeloperoxidase activity and the acid phosphatase's sensitivity to tartrate. They show a high level of morphological plasticity, as, upon stimulation with mitogens, they change morphology and obtain branching protrusions similarly to dendritic cells. We suggest, based on our findings, that the small, round cells described here are progenitor cells with potential to differentiate into dendritic like cells, although we can not exclude the possibility that they represent a novel cell type.

## Introduction

In fish, the phagocytic defense mechanism plays a pivotal role in non-specific immunity. As known, macrophages and neutrophils have high phagocytic activity. Among the small leukocytes, fish B-cells are professional phagocytes with microbicidal abilities [1], [2]. Phagocytosis by other professional phagocytic cells in fish like dendritic cells (DCs) have only recently been described [3], [4]. It has also been reported that fish thrombocytes are able to take up and kill *Staphylococcus aureus* [5], but it has not been established whether fish thrombocytes are capable of true phagocytosis, or the aforementioned antigen uptake was due to the open canalicular system in thrombocytes [6].

Teleost fish is the earliest evolutionary group having an adaptive immune system containing functional lymphocytes, immunoglobulin, T cell receptor (TCR), major histocompatibility complex and cytokines (reviewed in [7]) and equivalents of most of the cell types in mammals are present in fish, including lymphocytes, monocytes, macrophages and granulocytes. However, the research field of fish immunology is hampered by the lack of available tools, such as monoclonal antibodies, to isolate subpopulations of leukocytes. Because of this, there is currently limited knowledge about the cell specific markers, hematopoiesis and activation/maturation stages of cells, and identification of cell subsets in fish is often based on morphology. The exception is zebrafish were many hematopoietic genes are identified, and a more detailed picture of the development of the distinct leukocytes exists ([8], [9] reviewed in [10]). Gene expression analyses are a valuable tool for species for which the genome is sequenced, although there is currently little knowledge of the specific gene expression pattern in the different cell subsets in fish.

Until recently, it was unknown whether fish also have DCs. In mammals, DCs are essential immune cells bridging the innate and adaptive immunity. In their immature stage, the DCs have high antigen capture and processing ability, but their capability to prime T-cell responses is low. Upon activation, these actions are reversed, and the phagocytic activity decreases while their ability to present antigens to lymphocytes increases. DCs have a unique capability to activate naive T cells, in addition to memory T- and B-cells, making them the most potent of all antigen presenting cells (APCs) (reviewed e.g. in [11], [12]). Several recent reports from different research groups have described fish cells with DC-like features, strongly indicating at this cell type also is present in fish. Lovy and his coworkers performed immunohistochemistry of tissue using an antibody to human Langerin and showed cells with Birbeck granules [13], [14], [15]. Isolated cells with dendritic-like morphology and features have now also been reported. We showed immunostained cells with dendritic cell morphology containing long, branching protrusions using antiserum raised against the head kidney derived cell line, TO [16]. The observation that these cells were most abundant among spleen leukocytes is not surprising as spleen is the most prominent secondary lymphoid organ in fish and also, it was from spleen in mice Steinman and Cohn isolated DCs for the first time [17], [18], [19], [20], [21]. Lugo-Villarino and his coworkers identified and characterized an APC subset in zebrafish resembling mammalian DCs [3]. They enriched cells from whole kidney marrow with affinity for the lectin Peanut agglutinin, and they showed that these cells were able to stimulate T cells. DC-like cells have now also been isolated from several other organs in zebrafish [22]. Aghaallaei and his coworkers characterized mononuclear phagocytic cells with a dendritic phenotype isolated from transgenic medaka fish using the chemokine receptor cxcr3a as a marker [23]. Recently, Bassity and Clark described cells from trout with DC-like features, including morphology, motility and phagocytic ability [4]. These cells could be isolated directly from the spleen or derived from hematopoietic tissue and peripheral blood mononuclear cells *in vitro* [4]. These reports strongly support that DCs are indeed present in fish, but the knowledge of their hematopoietic origin, function and importance in the immune system has just started to emerge.

In mammals, the DCs represent a heterogeneous group of APCs consisting of several subpopulations with functional diversity, as well as diversification in surface markers and location in organs and tissue (reviewed in [11], [24]). The surface marker CD83, however, is a commonly used DC marker which is up regulated in mature DCs ([25], reviewed in [26]). There are indispensable interspecies differences between human and mice, both in surface markers and differentiation patterns [27], [28] and the complexity according to origin and diversification pattern of DCs are a highly debated issue [29]. The confusion of different cell types and morphologies is also true for fish leukocytes and thrombocytes. In contrast to thrombocytes in mammals, fish thrombocytes are nucleated cells. Their cell size and morphology are highly variable, including cells with ellipsoid, fusiform and spindle shape and oval or round cells [30], [31]. This variation might represent different maturation stages and/or different functional subtypes [32].

Except for the B-cells, the small mononuclear fish blood cells (<5 µm) are functionally poorly characterized, mainly due to the lack of reagents and suitable methods to isolate subpopulations. The aim of this study was to isolate and characterize a population of small salmon blood leukocytes using magnetic activated cell sorting, flow cytometry, functional assays, gene expression analyses and microscopy. We found that these cells expressed genes encoding the DC marker CD83 and MHC class II which is characteristic for APCs, but lack markers for all mature cell types investigated. Upon stimulation, these cells obtained dendritic morphology. Thus, based on the characterization of the cells presented in this study, we suggest that they are progenitor cells with potential to develop into DC like cells. The potent phagocytic ability suggest that these cells might have a role in innate immunity, and possible also forming a link to the adaptive part of the immune system due to the expression of MHC class II, but further investigation has to been performed for fully understand their role within the immune system.

## Materials and Methods

The present work with salmon has been conducted according to the approved national guidelines and performed according to prevailing animal welfare regulation. Rearing of fish under normal, optimal conditions does not require ethical approval under Norwegian law (FOR 1996-01-15 nr 23). All work in the presented manuscript has been done on cells isolated from dead fish. Fish were sacrificed with a sharp blow to the head, which is an appropriate procedure under Norwegian law.

### Fish and sampling procedure

Non-vaccinated, healthy Atlantic salmon (*Salmo salar* L.) smolts obtained from Urke Fiskeoppdrett AS, Norway, were kept in the wet lab, Bergen High-Technology center, under normal optimal rearing conditions at a temperature of 8°C, salinity of 34‰ and 24 hour light. These facilities are approved by the Norwegian Animal Research Authority for rearing of fish. The water flow was 1200 l per hour, and the fish was feed with commercial dry feed obtained from Skretting, Norway. There were no signs of infection and no mortality in the fish.

The fish were quickly netted and killed by a sharp blow to the head. Blood samples were collected as described below and stored on ice before further processing and fish length (cm) and weight (g) were recorded.

### Isolation of Peripheral Blood Leukocytes

Peripheral blood (4–8 ml) collected from vena caudalis of Atlantic salmon (*Salmo salar* L.), 600–1050 g of weight, using a syringe, was transferred to heparinised containers and diluted (1∶2) in Leibovitz L-15+ (L-15, (Cambrex) (adjusted to 370 mOsm by adding a solution consisting of 5% (v/v) 0.41 M NaCl, 0.33 M NaHCO3 and 0.66% (w/v) D-glucose), supplemented with 100 µg/ml gentamicin sulphate (Cambrex), 2 mM L-glutamine (Cambrex) and 15 mM HEPES). Leukocytes from peripheral blood (PBL) were then isolated on discontinuous Percoll gradients as described previously [33]. Cell number, aggregation factor and viability were analyzed using a CASY cell counter (Innovatis AG).

### Primary Antibodies

The polyclonal anti-TO serum used for immunofluorescence analysis stain all salmon leukocytes [16]. The Mab C4B6, reacting with different subtypes of leukocytes [33], [34], were used in magnetic activated cell sorting (MACS). For flow cytometry analyses, different monoclonal antibodies (MAbs) were used; C4B6 (anti-leukocytes), E3D9 (anti-neutrophils), C7G7 (anti-IgM/B-cells) and G2H3 (anti-IgM/B-cells). Their specificity and reactivity are described in [33], [34]. The MAbs 22-1, 42-1 and 8-3, reacting with trout thrombocytes [31], were a generous gift from Dr. Bernd Köllner, Friedrich-Loeffler-Institut, Germany. Also, a polyclonal Rabbit Anti-Human CD3 (DakoCytomation) was used in flow cytometry analyses. This antibody reacts with both T-cells and Ig+ cells in salmon (own unpublished analyses, earlier described as T-cell specific of Bakke-McKellep et al. [35]). A polyclonal anti-salmon IgM antibody (from our lab) was used in immunoblotting.

### Magnetic Activated Cell Sorting (MACS) of Salmon PBLs

PBL were fractioned with a MACS separation system (Miltenyi Biotec) using the MAb C4B6, reactive with salmon leukocytes, and goat anti-mouse IgG Microbeads (Miltenyi Biotec). PBL were incubated with 1 ml primary antibody solution (10 µg ml^−1^ C4B6-mouse IgG antibody in 1× PBS with 0.5% BSA per 2×10^7^ cells and maximum 3.5×10^7^ cells per LD column (Miltenyi Biotec)) for 20 min at 4°C and washed once with MACS buffer (1× PBS, 0.5% BSA and 2 mM EDTA, pH 7.3). Further, the cells were incubated with goat anti-mouse IgG Microbeads for 20 min at 4°C, washed once in MACS buffer, resuspended in 0.5 ml MACS buffer and filtered through a 40 µm nylon filter (BD Falcon). The cell suspension was then applied to a LD column and unmarked leukocytes (unbound fraction; termed C4B6^−^ throughout the text) and marked leukocytes (bound fraction; termed C4B6^+^ throughout the text) were collected according to the manufacturer's instructions. Following MACS separation, the cells were harvested and resuspended in Leibovitz L-15+ media. Cell number, aggregation factor and viability were analyzed using a CASY cell counter.

### Flow cytometry analyses of sorted cells

PBL and unbound fraction (C4B6^−^) after MACS separation was labeled with the MAbs C4B6, E3D9, C7G7, G2H3 and a polyclonal anti-human CD3 serum. The bound C4B6^+^ fraction was labeled with conjugate only. A MAb, 4D11, reactive with *Vibrio salmonicida* was used as isotypic control for the MAbs and rabbit preserum (Dako) was used as a negative control for the anti-human CD3 serum to define instrument settings for negative cells. Cells incubated without antibodies were used as control for auto fluorescence. R-phycoerythrin (RPE)-conjugated F(ab)_2_ –fragments of polyclonal rabbit anti-mouse (DakoCytomation) (1∶20) and RPE-conjugated F(ab)_2_ –fragments of goat anti-rabbit IgG (Molecular Probes, Invitrogen) (1∶200) was used as secondary antibodies and 10 000 cells were recorded in each sample. Cells were analyzed for forward scatter (FSC) and sideward scatter (SSC) patterns, representing the size and granularity of the cells, respectively, and for yellow fluorescence (FL2). Gating of the cells was performed to exclude dead cells, defined using propidium iodide (PI, sigma), and debris from subsequent analyses. Using the negative controls the cursor was set to 1% positive cells for MAbs and 2% for the polyclonal antiserum. The analyses were done on a FACSCalibur flow cytometer equipped with a 488 Argon laser using Cell Quest version 3.1 software (Becton Dickinson). Further data analyses were performed using FCS express 3 (De Novo Software). The experiments were performed with leukocytes from at least six fish.

### Flow cytometry assay of phagocytosis

Phagocytosis was studied using fluorescent latex beads and flow cytometry as described earlier [2] with some modifications. Five hundred µl cell suspension (1×10^7^ cells ml^−1^) were mixed with Fluoresbrite® YG carboxylate microspheres (Polysciences Inc.), 1 µm in diameter, at a cell: bead ratio of 1∶50 per well in 24 well cell culture plates (Nunc), and incubated for 4 hours at 15°C. Wells containing cell suspension without beads were used as negative controls. Following incubation with beads the cell suspensions were removed and the wells were washed once with 250 µl PBS 380 mOsm (PBS 380). Adherent cells were loosened by trypsination for 5 min using 250 µl per well of trypsin-EDTA (BioWhittaker) and gentle scraping. To remove non–ingested beads, the cell suspension was placed on top of a cushion consisting of 3 ml phosphate-buffered saline (PBS), pH 7.3, with 3% (w/v) bovine serum albumin (Sigma) and 4.5% (w/v) D-glucose, centrifuged at 100× g for 10 min at 4°C, washed once with 1 ml PBS+E (PBS containing 1% (w/v) BSA, 0.1% (w/v) sodium azide (Merck) and 25 mM EDTA (Merck)) and resuspended in 500 µl PBS+E prior to flow cytometry analyses.

Using flow cytometry, cells were analyzed for forward scatter (FSC) and sideward scatter (SSC) patterns, representing the size and granularity of the cells, respectively, and for green bead fluorescence (detected with 530/30 nm bandpass filter; FL1). Dead cells were detected by staining the cells with 2 µg ml^−1^ PI (using the 585/42 nm bandpass filter; FL2) and these cells were excluded from subsequent analyses by gating. The analyses were performed on a BD FACSCalibur flow cytometer equipped with a 15 mV 488 nm argon-ion laser using Cell Quest version 3.1 software (Becton Dickinson) recording 10 000 cells in each sample. Further data analyses were done using FCS express 3 (De Novo Software). Phagocytic ability was expressed as the percentage of total leukocytes with ingested beads, while the phagocytic capacity was expressed as the proportion of phagocytic cells that had ingested one, two, three or more beads. The experiments were performed with leukocytes from six fish.

### RNA isolation and qRT-PCR of magnetically sorted PBLs

Total RNA was prepared from 5×10^6^–1×10^7^ cells from the unbound C4B6^−^ fraction from four fish using GeneElute Mammalian Total RNA miniprep kit (Sigma) according to manufacturer's instructions and the quality and quantity was analyzed by OD_260/280_ and OD_260/230_ measurements using a NanoDrop® ND-1000 UV-Vis Spectrophotometer (NanoDrop Technologies). The RNA was treated with DNaseI (Sigma) to remove any traces of genomic DNA, quantified in the NanoDrop and further transcribed into cDNA using Taqman Reverse Transcription Reagents (Applied biosystems) according to the manufacturer's instructions, using random hexamers and 200 ng RNA in a 10 µl reaction. The synthesized cDNA samples were diluted 1∶10 in DNase- and RNase-free water (Sigma) and stored at −20°C.

qRT-PCR was performed with a MJ Research PTC-200 Peltier Thermal Cycler (BioRad VWR) using jumpstart Taq SYBRgreen kit for quantitative PCR (Sigma) and HPLC purified primers (Sigma-Genosys) shown in Table 1. qPCR assays made in this study were designed using Netprimer, Premier Biosoft. The primers were designed to span an exon-exon junction to eliminate signal from genomic DNA contamination. The PCR reactions contained 12.5 µl of 2× jumptart SYBRgreen (Sigma), cDNA (20 ng for the target gene and 2.5 ng for the reference gene EF1α), 0.25 µl ROX (diluted 10×), 1 µl (10 mM) of forward and reverse primers. The cycling conditions were 94°C for 5 min followed by 45 cycles of 94°C for 15 s and 60°C for 1 min. Two-fold serial dilutions of cDNA (20-1.25 ng) was made for efficiency calculations. Triplicate reactions of all the genes were performed. Negative controls without template (NTC) and RNA template (cDNA reactions without reverse transcriptase, -RT) were included for all master mixes. A melting curve analysis was performed following the real time PCR to ensure that a single amplicon was detected. In addition, amplified PCR products of all actual cDNAs were analyzed on a 2.5% agarose gel containing GelRed (Biotium), cloned into the pCR4-TOPO vector (Invitrogen) according to the manufacturer's instructions and sequenced to ensure that the correct cDNA sequences were amplified. The fragments were sequenced with BigDye version 3.1 fluorescent chemistry (Applied Biosystems) and run on an ABI PRISM® 377 DNA apparatus at the Sequencing Facility at the University of Bergen, Norway.

**Table 1:** Oligonucleotids used as primers in qRT-PCR.

Gene name	Acc.no (mRNA)	Forward primer (5′-3′)	Forward primer (5′-3′)	Amp size	Ref
EF1α	NM_001123629	CCCCTCCAGGACGTTTACAAA	CACACGGCCCACAGGTACA	57	[37]
mIgM	Y12457	GGTCCTTGGTAAAGAAACCCTACAA	CTGCATGGACAGTCAGTCAACAC	67	[62]
CD3ε	EF421420	TCAGGGCTCGGAAGAAGTCT	GCCACGGCCTGCTGA	68	[63]
CD8α	AY693393	CGTCTACAGCTGTGCATCAATCAA	GGCTGTGGTCATTGGTGTAGTC	119	[64]
TCRα	AY552002	GACAGCTACTACAGCCAGGTT	CAGAATGGTCAGGGATAGGAAGTT	126	[64]
MCSF-R	NM_001171807	GCAGAGGCCCAAATACTGC	TATGTCCAGCGACCAGGTG	73	[47]
CD86	DW580717.1	TAGACCACACACAGGGAACAATG	ATTGAGATGTATGTTCTTGTCGTCG	116	This study
CD83	BT047309	GCACCTGTAGGAGAGCAGAACC	TCCCTTTCTTCTGATTGGTCTGT	89	This study
MHCII	X70165	GTGGAGCACATCAGCCTCACT	GACGCACCGATGGCTATCTTA	92	[47]
GATA-1	NM_001139767	CCAACCTCCCTTTATGCCTCCA	GATTCAGAAAACTGCTTCCACCA	163	[50] This study
G6F	GU393010.1	GGGTCGCTACTCCTGTCTGATT	GATTCAGAAAACTGCTTCCACCA	94	This study

Elongation factor 1α was chosen as reference gene based on results from previously studies [36], [37].

### Cytospin preparations

Cytospin preparations of isolated leukocytes were prepared by centrifugation of 100 µl of cell suspension of 1×10^6^ cells ml^−1^ at 1000 rpm, medium acceleration for 3 min, using a Shandon Cytospin III cytocentrifuge. The cytospin preparations were air dried for 20 h at room temperature prior to Diff Quick-, immunofluorescence- or cytochemical staining.

### Immunofluorescence staining

Cytospin slides were fixed in acetone: methanol (1∶1) for 90 s, washed once in PBS, pH 7.3 and incubated in a humidity chamber in the dark with Immage-iT Fx signal enhancer (Molecular Probes, Invitrogen) for 30 min to block background staining. Further, the slides were incubated for 1 h in a humidity chamber in the dark with primary antibody (rabbit anti-TO, diluted 1∶1000 in PBS containing 0.5% (w/v) BSA), washed twice in PBS, pH 7.3 bath, incubated with Alexa Fluor 488 F(ab′)_2_ fragments of goat anti-rabbit IgG (5 µg ml^−1^) (Molecular probes, Invitrogen) for 45 min and washed 3 times in PBS, pH 7.3 bath prior to mounting with cover glass. When double immunostaining with anti-TO (1∶1000) and C4B6 (2 µg ml^−1^) was performed, the primary antibodies were mixed and so also the conjugates (Alexa Fluor 488 F(ab′)_2_ fragments of goat anti-rabbit IgG (5 µg ml^−1^) and Alexa Fluor 555 F(ab′)_2_ fragments of goat anti-mouse IgG (5 µg ml^−1^) (Molecular probes, Invitrogen). The slides were coverslipped with ProLong® Gold Antifade Reagent with DAPI (Molecular Probes). For negative controls, the primary antibody was omitted or rabbit preserum/4D11 was used as primary antibody. The experiment was performed with leucocytes from two fish and repeated five times.

### Respiratory burst assay

The respiratory burst assay used in this study was established by Kalgraff et al. [38] and is based on oxidation of dihydrorhodamine 123 (DHR) to the fluorescent rhodamine 123 (RHO). Both C4B6^−^ and C4B6^+^ fraction after MACS were diluted to a leukocyte concentration of 2.5×10^6^ ml^−1^ in PBS380 containing heparin (PBS380h). Two hundred µl was transferred to 5 ml polystyrene tubes (Falcon, Becton Dickinson) and incubated at 20°C for 10 min with gentle tilting. Respiratory burst activity was activated by Phorbol 12- myrisate 13- acetate (PMA) (Sigma) using a concentration of 1 µg ml^−1^ in the tube and incubated for 10 min at 20°C before addition of DHR. Five µl of 206 µM DHR, resulting in a total concentration of 5 µM per sample tube was added. The samples were mixed by careful vortexing and incubated with gently tilting for 30 min. Three hundred µl PBS380h was added to each sample and 100 µl were cytospun as described above. The cytospin preparations were immediately examined using fluorescence microscope Zeiss Axioskop 2 plus. Two controls were performed; one untreated sample without and PMA and DHR and one non-stimulated sample without PMA. The experiment was performed with MACS separated leucocytes from two fish and repeated three times.

### Myeloperoxidase (MPO)

The cytospin preparations were incubated in freshly prepared fixative solution (10% (v/v) 37% formaldehyde, 90% (v/v) 95% ethanol) for 30 s, washed in gently running tap water and air dried in the dark for 10 min. The preparations were then incubated with diaminobenzidine (DAB) staining solution prepared from SIGMA*FAST* DAB tablets (Sigma) for 30 min in the dark, rinsed in tap water and mounted in Glycergel mounting medium (DAKO). Staining was performed at room temperature. The staining was performed with MACS separated leucocytes from four fish.

### Acid Phosphatase

The acid phosphatase kit was obtained from Sigma and the staining was performed on cytospin slides following the manufacturing instructions. Two staining solutions were prepared, one with and one without tartrate solution. The staining was performed with MACS separated leucocytes from four fish.

### Mitogen stimulation

C4B6^−^ cells were left over night (12–16 hrs) at 15°C allowing the cells to recover after MACS. To each well in a 24 well-plate (Nunc), 1×10^6^ cells were added in L-15+ medium containing 50% TO cell culture supernatant, 10% fetal calf serum and without or with mitogens; Concanavalin A (Con A) lectin from *Canavalia ensiformis* (10 µg ml^−1^) (Sigma) and PMA (5 ng ml^−1^) or lipopolysaccharides (LPS) from *Escherichia coli* (500 µg/ml) (Sigma) in a total volume of 1 ml per well. The cells stimulated with Con A and PMA was incubated for 18 hrs at 15°C, while the LPS stimulated cells were incubated for 24 hrs at 15°C. Samples without mitogens were used as negative controls. The experiment was performed with leucocytes from two fish and repeated at least ten times.

### SDS polyacrylamide gel electrophoresis (PAGE) and Immunoblotting

PBL, unbound and bound fractions after MACS were fractioned on a 12% SDS-PAGE using a Mini Protean Tetra Cell (Biorad). Samples of 12 µl (5×10^5^ cells) were loaded onto each well, electrophoresed at 190 V for 45 min, followed by staining of proteins (Silver Stain Pluss, BioRad) or electroblotting onto a nitrocellulose membrane. Immunoblot analysis was performed using anti-salmon IgM polyclonal antibody (1∶1000) and peroxidase-conjugated goat anti-rabbit IgG (BioRad) (1∶3000) and developed using enhanced chemiluminescence (Thermo Scientific Pierce). The experiment was performed with PBL and MACS separated leucocytes from three fish.

### Transmission Electron Microscopy (TEM) and Scanning Electron Microscopy (SEM)

C4B6^−^ cells from a phagocytosis assay using 1 µm Fluoresbrite® YG carboxylate microspheres (Polysciences Inc.) or Polybead polystyrene 2 µm microspheres (Polysciences Inc.) were fixed in 1.5–2% glutaraldehyd in 0.1 M sodium cacodylate buffer following postfixation in 1% osmium tetraoxide in 0.1 M sodium cacodylate buffer. The TEM samples were embedded in Agar 100 Resin and thin sections (50–60 nm) were stained with uranyl and lead. The samples for SEM analyses were treated as described above, but after postfixation the cells were transferred to coated cover slips. TEM and SEM samples were analyzed at the Molecular Imaging Center (Fuge, Norwegian Research Council), University of Bergen, using Joel JEM-1230 (transmission electron microscope) and Jeol JSM-7400F (scanning electron microscope). The experiment was performed with C4B6^−^ cells isolated from two fish.

### Digital imaging and image processing

All slide preparations, except for those used in electron microscopy, were examined in a Zeiss Axioskop 2 plus and photographs of the cells were captured using a Nikon DS camera Head DS-5M. The pictures and the overlays were prepared by processing in Adobe Photoshop CS5.

## Results

### Double immunostaining with anti-TO and MAb C4B6 revealed small, round C4B6^−^ cells which are abundant among PBL

**Figure 1:**
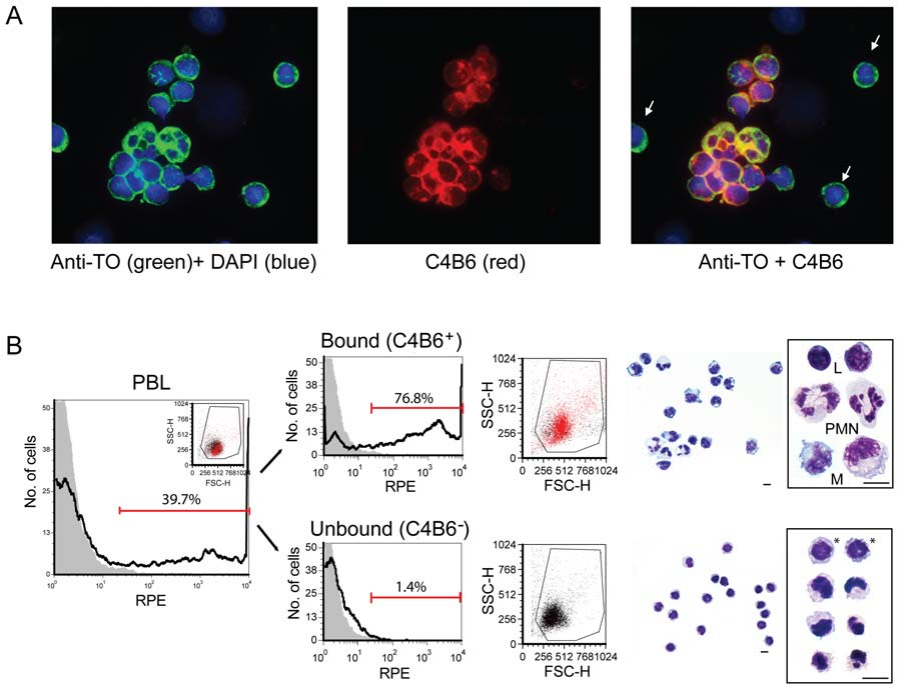
C4B6^−^ cells are small, round cells which are abundant among the PBL. (A) Double immunostaining with anti-TO (green) and MAb C4B6 (red) revealed small, round cells that were C4B6^−^ (white arrows). (B) Flow cytometry histograms of PBL prior and after MACS separation. The bound fraction (C4B6^+^ cells) contained both polymorphonuclear (PML) and mononuclear cells (L = leukocytes, M = monocytes) while the cells in the unbound fraction (C4B6^−^ cells) were small, mononuclear cells with low level of granularity. Cells with large, round nuclei were present in most C4B6^−^ cells (labeled with *), although cells with other nuclei forms were observed. C4B6^+^ cells are shown as red dots in the dot plots (middle panels). The markers define the C4B6^+^ cells. Diff Quick stained cells are shown in the right panels. Representative single cells are enlarged. The diff quick pictures are captured with 63× objective. Scale bar = 5 µm.

Double immunostaining of cytospin preparations of PBL with the polyclonal anti-TO antibody, staining all salmon leukocytes, and the MAb C4B6, reacting with different cell types like polymorphonuclear (PMN) leukocytes, B-cells and monocytes/macrophages, revealed small, round cells that were C4B6^−^ (Fig. 1). The C4B6^−^ cells were highly abundant among the PBL (55.8%±6.8%, n = 5, Fig. 1B and [34]). In order to isolate these cells for further characterization, magnetic activated cell sorting were performed using MAb C4B6 and magnetic beads. The depleted, unbound fraction consisted mainly of small, round cells (Fig. 1B, Fig. S1A, B) with low granularity as seen in the scatter plot (Fig. 1B). Also, thrombocytes with oval, fusiform or spindle morphology were observed among them. The bound fraction consisted mainly of PMNs, monocytes and lymphocytes (Fig. 1B), although a fraction of C4B6^−^ cells were present, as expected, as the protocol was optimized to avoid C4B6^+^ cells in the unbound fraction. In the unbound fraction, the purity of the C4B6^−^ cells was 97.5±1.5%. The outcome of C4B6^−^ cells were 23.9%±7.3% (n = 31) and the % viability was 96.0%±1.2% (n = 29).

### SEM and TEM revealed morphological heterogeneity

The majority of the C4B6^−^ cells were small (<5 µm) and round, but the nuclear shape varied from round (most common) to oval, bean and irregular-shaped (Fig. 1B, lower right panel, Fig. S1A, B). The nucleus to cell ratio in most cells were high as for leukocytes. To study the cell morphology of the C4B6^−^ cells in more detail, transmission and scanning electron microscopy were performed (Fig. S1C, D). Ultra structural analysis revealed that the cells had a densely packed nucleus and some of the cells had vacuoles and/or granules in the cytoplasm (Fig. S1C). A typical thrombocyte is shown in Fig S1E for comparison. SEM analysis showed that some of the cells had smooth surface with crater like depressions, while others had a crumbled surface with folds (Fig. S1D). Also, some flat oval cells, likely precursors of erythrocytes, and thrombocytes were seen (Fig. S1F).

### The C4B6^−^ cells lack markers for B-cells, T-cells, monocytes, macrophages and neutrophils, but express genes encoding CD83 and MHC class II

**Figure 2:**
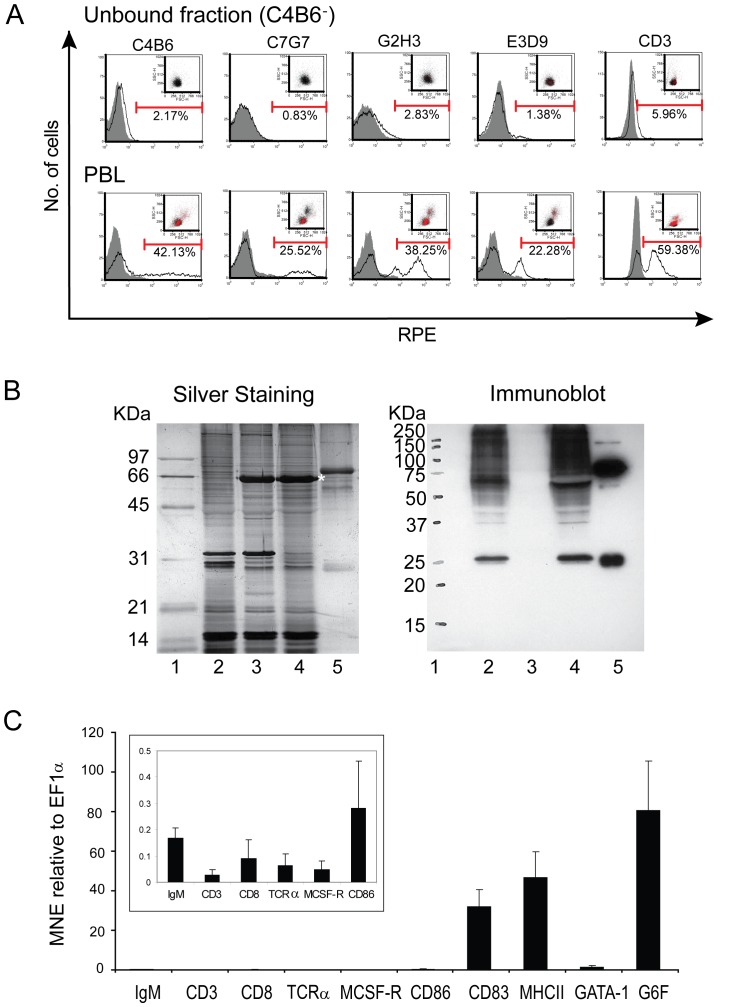
The C4B6^−^ cells are negative for lymphocyte-, neutrophil and monocyte/macrophage markers, but express CD83 and MHC class II. (A) Flow cytometry analyses of the unbound fraction after MACS show the C4B6^−^ cells' reactivity with MAb C4B6 (anti-leukocytes), C7G7 and G2H3 (B-cells), E3D9 (neutrophils) and the polyclonal anti-human CD3 antiserum (T- and IgM^+^ cells). Representative histograms are shown. Grey filled curves are negative controls. Positive cells are shown as red dots. The markers represent positive cells. The antibodies reactivity against PBL prior to MACS is shown in the lowest panels. (B) A silver stained SDS- polyacrylamide gel, left panel, and an immunoblot using polyclonal IgM serum developed with ECL, right panel. Lane 1, molecular mass markers; lane 2, PBL; lane 3, unbound fraction after MACS (C4B6^−^ cells); lane 4, bound fraction after MACS (C4B6^+^ cells); lane 5, salmon IgM. The 66 KDa band (*) in lane 3 and 4 is most likely BSA present in the MACS buffer. (C) qRT-PCR analysis of the C4B6^−^ cells. Gene expression of the following genes; IgM (B-cell marker), CD3, CD8 and TCRα (T-cell markers), MCSF-R (monocyte/macrophage marker), CD86 (involved in antigen presentation), CD83 (DC marker), MHC class II (APC), GATA-1 and G6F (thrombocyte/erythrocyte markers) is presented as mean normalized expression (MNE) using EF1α as reference gene (n = 4). The average of triplicates from four fish with standard error is shown. Note different scale in the inserted histogram.

In order to characterize these small, round C4B6^−^ cells, different methods were used. All currently available antibodies against salmon leucocytes were used to label the cells and analyze them with flow cytometry. As shown in Figure 2A, the C4B6^−^ cells lack the C4B6 epitope and they did not react with antibodies against B-cells (MAb C7G7, MAb G2H3), neutrophils (MAb E3D9) and T- cells (PAb CD3). The CD3 antibody reacts with Ig^+^ in salmon (our unpublished data) in addition to presumably T-cells, as suggested earlier by Bakke–McKellep [35]. Previous publications have reported the above mentioned MAbs to salmon leucocytes [33], [34]. However, to ensure that the lack of binding was not a result of the experimental design, the PBL fractions prior to magnetic activated cell sorting (MACS) were included as positive controls (Fig. 2A, lower panels). The antibodies reactivity 42.13% (C4B6), 25.26% (C7G7), 38.14% (G2H3), 22.0% (E3D9) were in accordance with previous studies and 59.38% CD3 positive cells are similar to other studies in our lab (unpublished data). Studies from trout and salmon have shown that B-cells, which also have a small, round morphology, have potent phagocytic activity. To ensure that the C4B6^−^ cells were not B-cells, immunoblot analysis was performed using a polyclonal IgM antibody against B-cells (Fig. 2B). Included in our analyses were unsorted PBLs, and the unbound and bound fraction from MACS. Also, purified salmon IgM was included as a positive control. The immunoblot analysis showed that the unbound fraction did not react with the IgM antibody and thereby confirming the flow cytometry results. In our next step to identify these cells, qRT-PCR was performed (Fig. 2C). Interestingly, the CD83 gene, which is the hallmark for DCs in mammals and also present on DCs in teleosts, was highly expressed. Further, MHC class II which is limited to professional antigen-presenting cells was also abundant. The C4B6^−^ cells did not express genes characteristic for B- cells (IgM), T- cells (CD3, CD8 and TCRα) or monocytes/macrophages (MCSF-R). The minute expressions of the aforementioned genes, shown in Figure 2C, inserted histogram, are probably due to background levels of the other cell types. Also, CD86 was expressed, although at a very low level. In addition, the genes GATA-1 and G6F specific to thrombocytes and erythrocytes were expressed.

### The C4B6^−^ cells have potent phagocytic activity

**Figure 3:**
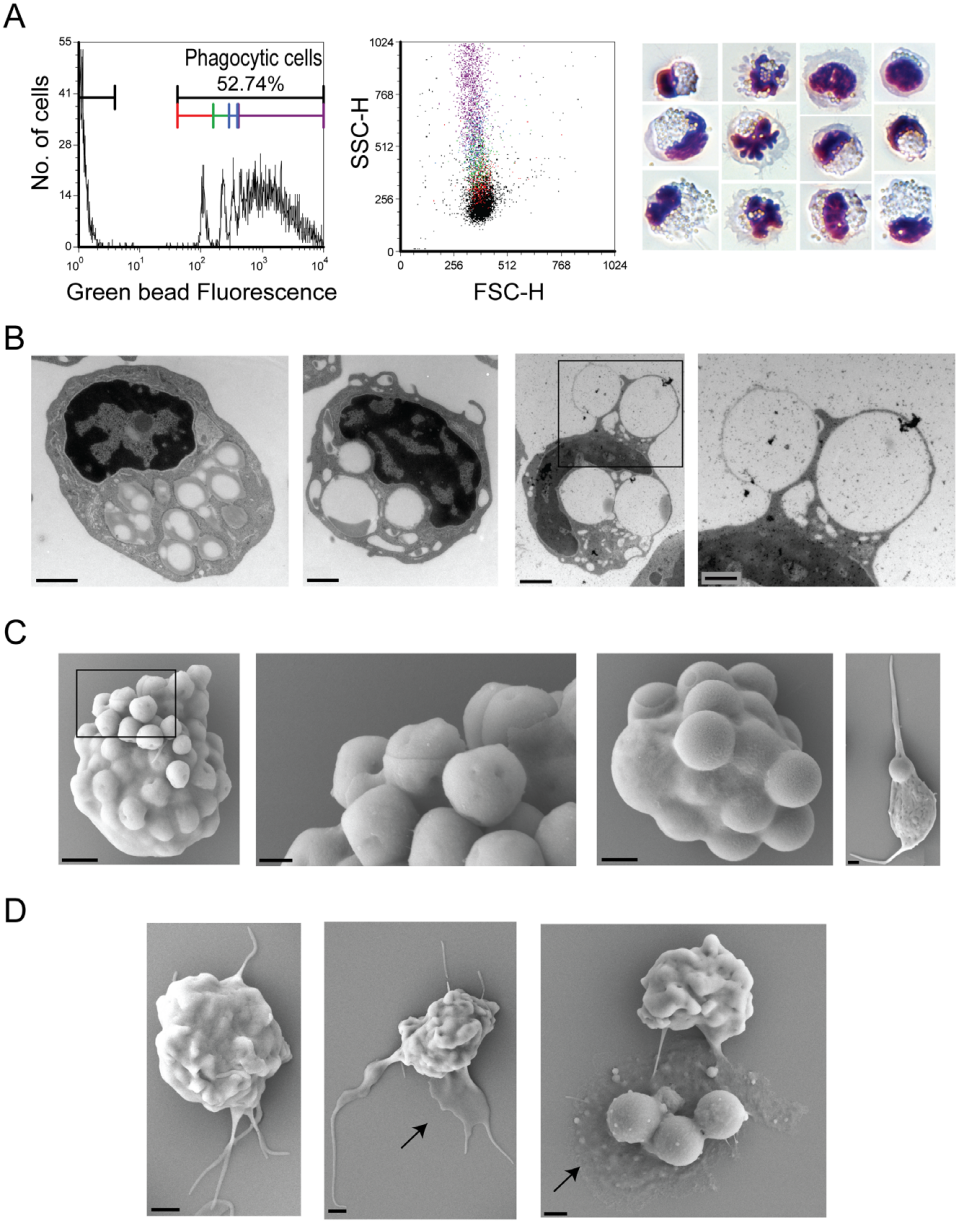
The C4B6^−^ cells have potent phagocytic activity. (A) A representative histogram showing the phagocytic activity (left panel) of the C4B6^−^ cells. Increased peak fluorescence indicates an increased number of ingested beads. The colors of the markers reflect the colors in the dot plot (middle panel). The dot plot shows the distribution of cells with various number of cells; cells without beads are shown as black dots, cells with one bead are red, cells with two beads are green, cells with three beads are blue and cells with more than three beads is shown as purple. Right panel, Diff Quick stained cells with various numbers of ingested beads. (B) TEM analyses of C4B6^−^ cells with beads. Left panel, a representative cell containing several 1 µm beads (magnification = 20 000×, scale bar = 1 µm). Middle panels, cells containing beads that are 2 µm (magnification = 15 000×, scale bar = 1 µm). Right panel is an enlargement of the delimited area of the middle, right panel showing beads that are just about to be ingested (magnification = 30 000×, scale bar = 0.5 µm). (C) SEM of C4B6^−^ cells ingesting beads that are 1 µm (two left panels) and 2 µm (two right panels). Notice the cell membrane that is about to enclose around the beads. A typical thrombocyte, containing one 2 µm bead, is shown in the right panel for comparison. In panels from left to right: Magnification (scale bar); 10 000× (1 µm), 30 000× (0.3 µm), 10 000× (1 µm) and 5000× (1 µm). (D). Different stages of bead capturing. Notice the veil (arrow). In panels from left to right: Magnification (scale bar); 8000× (1 µm), 5000× (1 µm) and 6500× (1 µm).

As the qRT-PCR analyses revealed that the C4B6^−^ cells expressed MHCII which is limited to professional antigen-presenting cells, we investigated their phagocytic ability and killing/degrading mechanism(s) as these actions are prerequisites to antigen-presentation. A substantial fraction of the C4B6^−^ cells had taken up fluorescent beads (52.7%±16.0%) and most of the phagocytic cells had high capacity as they had taken up 3 or more beads (Fig. 3A middle and right panel). The granularity of the cells increases with increased number of phagocytized particles (Fig. 3A middle panel). Diff Quick cytospin preparations of the C4B6^−^ fraction show cells with different cell and nucleus morphology (Fig. 3A, right panel), but it has to be noticed that the cell shapes might be affected by the numerous beads inside some of the cells. The fluorescent beads appear white in the light microscopy. To ensure the beads were not simply attached to the cell surface, but actually internalized in the cells, TEM and SEM analyses were performed of cells after incubation with beads of different sizes (Fig. 3B and C, respectively). The two most and second left panels in Figure 3B show TEM pictures of representative cells ingested 1 µm and 2 µm beads, respectively. The two panels to the right show a phagocytic cell that is about to ingest two 2 µm beads. Two other beads (2 µm) are already ingested. The high phagocytic capacity of the C4B6^−^ cells are shown by SEM of representative cells (Fig. 3C). The two panels to the left show a representative cell digesting 1 µm beads, the cell in the second right panel have engulfed numerous beads that are 2 µm in size. Notice the nucleus that is pointing out towards the left side of the cell. A SEM picture of a typical thrombocyte is shown in Figure 3C, right panel, for comparison. A suggested strategy for capturing foreign material include veils, as shown by SEM of representative cells (Fig. 3D).

### The C4B6^−^ cells use an oxygen independent killing mechanism

**Figure 4:**
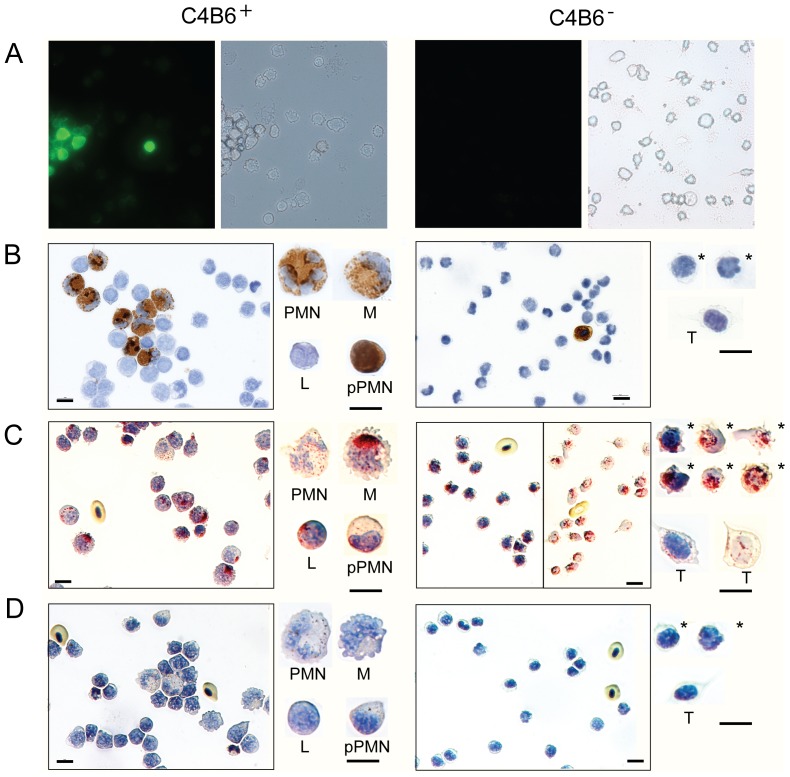
The C4B6^−^ cells have an oxygen-independent mechanism. Panels to the left, C4B6^+^ cells, are included as positive controls. Panels to the right, C4B6^−^ cells. (A) Respiratory burst activity. Cells were stimulated with PMA and positive cells appear green in fluorescence microscopy due to RHO123 fluorescent (left panels). The corresponding bright field photos are shown in the right panels. Magnification: 400×. (B) Myeloperoxidase activity. Positive cells appear brown while cells without activity are blue. (C) Acid phosphatase staining. Granular sites of activity appear red. For the C4B6^−^ cells, notice that the overview picture to the right has less counterstaining, for easier observation of the positive granules. (D) Tartrate resistant acid phosphatase staining. Granular sites of activity appear red. Representative single cells are enlarged; PMN = polymorphonuclear, M = monocytes, L = leukocytes,* = representative C4B6^−^ cells, T = thrombocytes. Pictures in B–D are captured with 63× objective. Scale bar = 10 µm.

To degrade/kill foreign particles and microbes, the cells can use an oxygen dependent strategy (respiratory burst, myeloperoxidase) or a strategy independent of oxygen (hydrolytic enzymes, such as acid phosphatase). As shown in Figure 4, the C4B6^−^ cells did not have respiratory burst or myeloperoxidase (MPO) activity (Fig. 4A, B), but they were positive for acid phosphatase staining (Fig. 4C). Included as controls is the bound fraction after MACS (left panels in Fig. 4). The bound fraction (C4B6^+^) contains, among other, neutrophils that are known to have strong respiratory activity and are positive for MPO. Monocytes can also have MPO activity, but not lymphocytes. The acid phosphate in the C4B6^+^ cells was sensitive to tartrate (Fig. 4D), supporting an oxygen-independent killing mechanism. Neutrophils have both tartrate-resistant and sensitive acid phosphatases (Fig. 4C, D)

### The cells change morphology upon mitogen stimulation

**Figure 5:**
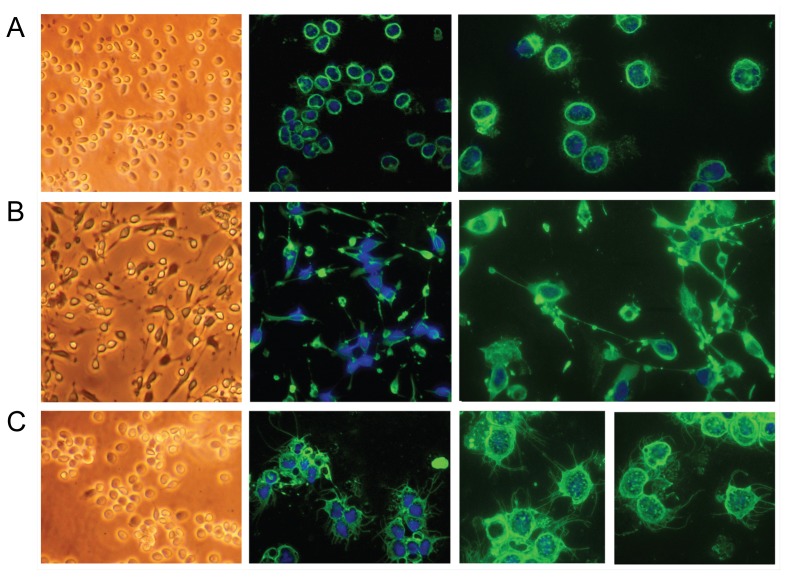
The C4B6^−^ cells change morphology upon stimulation with various mitogens. Left panels, inverted microscope pictures. Middle panels, fluorescence microscopy pictures of cells stained with anti-TO antiserum, captured with 40× objective. Right panels, fluorescence microscopy pictures of cells stained with anti-TO antiserum, captured with 63× objective. (A) Cells without mitogen, 24 hrs incubation. (B) Cells stimulated with Con A and PMA for 18 hrs. (C) Cells stimulated with LPS for 24 hrs.

Upon stimulation with different mitogens, the C4B6^−^ cells changed morphology (Fig. 5). In the presence of Con A and PMA, these normally non-adherent cells became adherent with a few, very long protrusions while in the presence of LPS the cells got many, thin protrusions, some branching, emanating from all of the cell body. The LPS stimulated cells were non-adherent. It has to be noted that TO cell culture supernatant, which likely contain different cytokines, was added to all samples. However, the supernatant without mitogen did not cause changes in cell morphology (Fig. 5A).

## Discussion

Differentiation between the subsets of leukocytes is not obvious, and it is becoming clear that mammalian monocytes, macrophages and DCs are not homogenous populations as they can differentiate into discrete functional subsets (reviewed in [39]). Also, the high plasticity in surface marker expression of the cells within the mammalian mononuclear phagocytic system, cause difficulties when differentiating between developmental stages and cell subsets (reviewed in [40], [41]). The knowledge of the different cell types and hematopoiesis of the cells in teleosts has, in addition been hindered of few, available antibodies against different cell types, limited information about the specificity of cell markers and also, as yet, only relatively few sequenced fish genomes are available. Therefore, the studies on fish cells often base on knowledge of the mammalian systems, which are far more investigated. This is despite the fact that there are crucial differences between mammals and teleosts; for example the fish lack lymph and lymph nodes and their thrombocytes and erythrocytes are nucleated cells. Also, due to the poikilothermic nature of the fish, the innate immune system is likely of greater importance compared to mammals.

Salmon, like other fish species, have equivalents of most of the mammalian cells; lymphocytes (B- and T-cells), monocytes/macrophages, granulocytes (neutrophils, basophils and eosinophils) and non- specific cytotoxic cell which are the counterpart to mammalian NK cells. Also, cells with dendritic like morphology and characteristics have recently been reported [13], [14], [15], [16]. The available antibodies for subtypes of viable salmon cells are C7G7, G2H3 and IgM (reacting with B-cells), E3D9- (reacting with neutrophils) and C4B6 (reacting with several different leukocytes) and anti-TO antibody reacting with all salmon leukocytes. Combining the two latter mentioned antibodies, immunostaining of PBL revealed some small, round cells that were C4B6^−^. These cells were abundant among the PBL. We isolated them for further characterization and identification. Phagocytosis assay revealed that these cells had a remarkable high phagocytic capacity, indicating that these cells are professional phagocytes. Many phagocytes produce reactive oxygen species with antimicrobial effect in a process called respiratory burst to degrade internalized microbes. Alternatively, pathogens can be degraded in an oxygen-independent mechanism using hydrolytic enzymes like acid phosphatase. The C4B6^−^ cells isolated in this study stained strongly positive in the acid phosphatase assay similarly to B-cells in trout [1]. Absent respiratory burst and myeloperoxidase activity and the acid phosphatase's sensitivity for tartrate confirmed an oxygen independent killing mechanism. Tartrate-resistant acid phosphatase is part of the respiratory burst process, due to the presence of a redox active iron which catalyses the generation of reactive oxygen species through Fenton chemistry [42], and only presents in neutrophils and their precursor cells.

Using different approaches, we characterized the C4B6^−^ cells. Gene expression study showed that these cells were not lymphocytes as they did not express the genes IgM (specific for B-cells) or TCRα, CD3 and CD8 (specific for T-cells). Flow cytometry analyses and immunoblotting supported these findings. Further, the C4B6^−^ cells neither express the gene encoding MCSF-R nor react with anti-neutrophil antibody. Interestingly, they did express the gene encoding CD83 which is a hallmark for DCs in mammals ([25], reviewed in [11], [43]). It should be noted that activated lymphocytes in mammalian also express CD83 [44]. The exact function of CD83 is not yet fully understood, but surface expression of CD83 is positively correlated to CD86 and MHC class II expression and likely involved in T- and B- lymphocyte maturation [26]. The presence of CD83 in the different cell subsets in teleosts is not well known, but DCs isolated from rainbow trout express CD83 [4]. CD83 is, however, also present in trout macrophages [45], [46] and different cell lines like the highly phagocytic salmon TO cell line [47], the salmon pronephros-derived mononuclear leukocyte (SHK-1) cell line [48] and in the macrophage –like cell line RTS11 from trout [49]. The CD86, a costimulatory molecule expressed on APCs which provides signals necessary for T cell activation, is present in low levels in immature DCs, as in the C4B6^−^ cells, but is highly expressed in mature DCs [11].

The C4B6^−^ cells isolated in this study also express MHC class II limited to professional antigen presenting cells. In addition, qRT-PCR analyses showed expression of genes limited to thrombocytes/erythrocyte linage, G6F [50] and GATA-1, as expected as thrombocytes could be observed in the C4B6^−^ fraction and precursor cells for erythrocytes are likely found in blood. In zebrafish and mammals, GATA-1 drives the differentiation of hematopoietic progenitors into a subset of the blood cell lineages including erythrocytes, megakaryocytes, eosinophils, and mast cells ([51], reviewed in [52]).

Are the C4B6^−^ cells progenitor cells or a new cell type? Based on the presented data, there are several options according to the identity of these cells. They are not lymphocytes as shown by flow cytometry, qRT-PCR and immunoblotting. They are not either neutrophils or monocytes/macrophages due to flow analyses, qRT-PCR and functional analyses like respiratory burst and myeloperoxidase activity. Also, they were absent for tartrate resistant acid phosphatase which are part of the oxygen-dependent degrading mechanism found in neutrophils. However, we can not exclude the possibility that they are progenitor cells with potential to differentiate into one or more of the aforementioned cell types. Morphologically, they are small, round cells different from typical thrombocytes with characteristic oval, spindle, spiked or fusiform shape. TEM analyses revealed, however, that many of the C4B6^−^ cells had vacuolated cytoplasm similar to thrombocytes suggesting that these cells might be related/progenitors to thrombocytes. As far as we know, megakaryocytic erythrocyte progenitor or precursor cells with both thrombocytes/erythrocyte and myeloid potential (common myeloid progenitor) [39] in fish has not been characterized at an ultra structural level. Also, following MACS separation, a substantial portion of the C4B6^−^ cells reacted with two of three tested antibodies against trout thrombocytes (data not shown). Therefore, we can not exclude the possibility that these cells are progenitor cells with thrombocyte potential or a thrombocyte subset not previously described. They are different to “classical” thrombocytes functionally, both with regards to the phagocytic ability and capacity and also the intensity of the acid phosphatase staining. The small, round cells were highly positive with intense red staining due to numerous granules in the cytoplasm while the typical thrombocytes were only faintly or not positive. The MAb 22-1, 8-3 and 42-1, which were all involved in thrombocytes aggregation induced by collagen, recognized 17 and 21 kDa surface proteins [31]. However, the exact epitope for the anti-thrombocytes trout MAbs are not yet known, and there are several examples of cell surface markers that are common for multipotent hematopoietic progenitors and thrombocytes, like thrombomucin [53]. *In vivo*, several cell types reside in direct proximity to extracellular matrix proteins and one of these proteins, collagen, affect morphology and function, involved in activation and/or differentiation, of a number of cell types like neutrophils and DCs [54], [55], in addition to thrombocytes. It is crucial to bear in mind that the classification of different cell types in fish, up till now, is often performed at a morphological basis alone, due to lack of specific cell markers and antibodies. Few studies of hematopoiesis of different cell types and progenitor cells in fish are available. Also, many morphological analyses were performed prior to the discovery of DCs in fish, which shed new light on the leucocytes differentiation in fish. Immune cell development in fish is interesting for basic research and from a phylogenetic point of view as fish is the first evolutionary group with adaptive immunity. As such, knowledge of the ontogeny of immune cells in fish can provide valuable information about the origin of mammalian immune cells and immune system. Furthermore, knowledge of the immune system in salmon has impact on the aquaculture industry as it one of the most important farmed fish in Norway and worldwide.

The presence of CD83, predominantly expressed on DCs, together with the expression of MHC class II, the high phagocytic potential which is typically for immature DCs and the ability to change morphology upon stimulation are all features similarly to immature DCs. We suggest, therefore, that the C4B6^−^ cells are progenitor cells with potential to differentiate to DCs, although we cannot exclude the possibility that they represent a novel cell type. Mononuclear progenitors are also present in human and mice blood, which can, upon stimulation, differentiate into DCs [56], [57], [58], [59], [60]. The morphology of C4B6^−^ cells are similar to common dendritic cell precursor (CDP) and CDP-derived circulating DC precursors with pDC and cDC potential [60]. Small, phagocytic IgM^−^ cells that are not T-cells have also been reported from trout [1]. A possible novel cell type consisting of small, round cells in Murray Cod [61] have similar cytochemical features as the C4B6^−^ cells and also as fish DCs [3]. Based on the initial characterization of the C4B6^−^ cells, presented in this paper, we can only speculate about their exact immune functions. Further in-depth analyses of the stimulated C4B6^−^ cells will provide better knowledge to fully understand their roles in immunity. It is, however, likely that the C4B6^−^ cells have a significant role in antigen trapping because of their potent phagocytic capacity and possible also antigen-presentation due to the presence of CD83 and MHCII. The DC potential of the C4B6^−^ cells has to be investigated further as DC progenitors will be valuable tools to elucidate the hematopoiesis and function of the newly discovered DCs in fish.

## Supporting Information

Figure S1 **Morphological analyzes of a suspension of purified C4B6^−^ cells using Diff Quick staining, immunostaining and electron microscopy.** (A) Diff Quick staining (magnification 630×, scale bar = 5 µm). (B) Immunostaining using the polyclonal anti-TO antiserum (magnification 630×, scale bar = 5 µm). (C) TEM of representative cells (magnification left panel: 5 000×, right panels: 20 000×, scale bar = 1 µm). (D) SEM of representative cells (magnification 10 000×, scale bar = 1 µm). (E) TEM of a typical thrombocyte is shown for comparison (magnification 15 000×, scale bar = 1 µm). (F) SEM of an erythrocyte precursor, upper panel (magnification 10 000×, scale bar = 1 µm) and a typical thrombocyte, lower panel (magnification 5 000×, scale bar = 1 µm) is shown for comparison.
(TIF)
