# Supplementary figures and images for: Characterization of Small, Mononuclear Blood Cells from Salmon Having High Phagocytic Capacity and Ability to Differentiate into Dendritic like Cells
Source: PLoS One. 2012 Nov 14;7(11):e49260. doi: 10.1371/journal.pone.0049260 (PMC3498127; doi:10.1371/journal.pone.0049260)

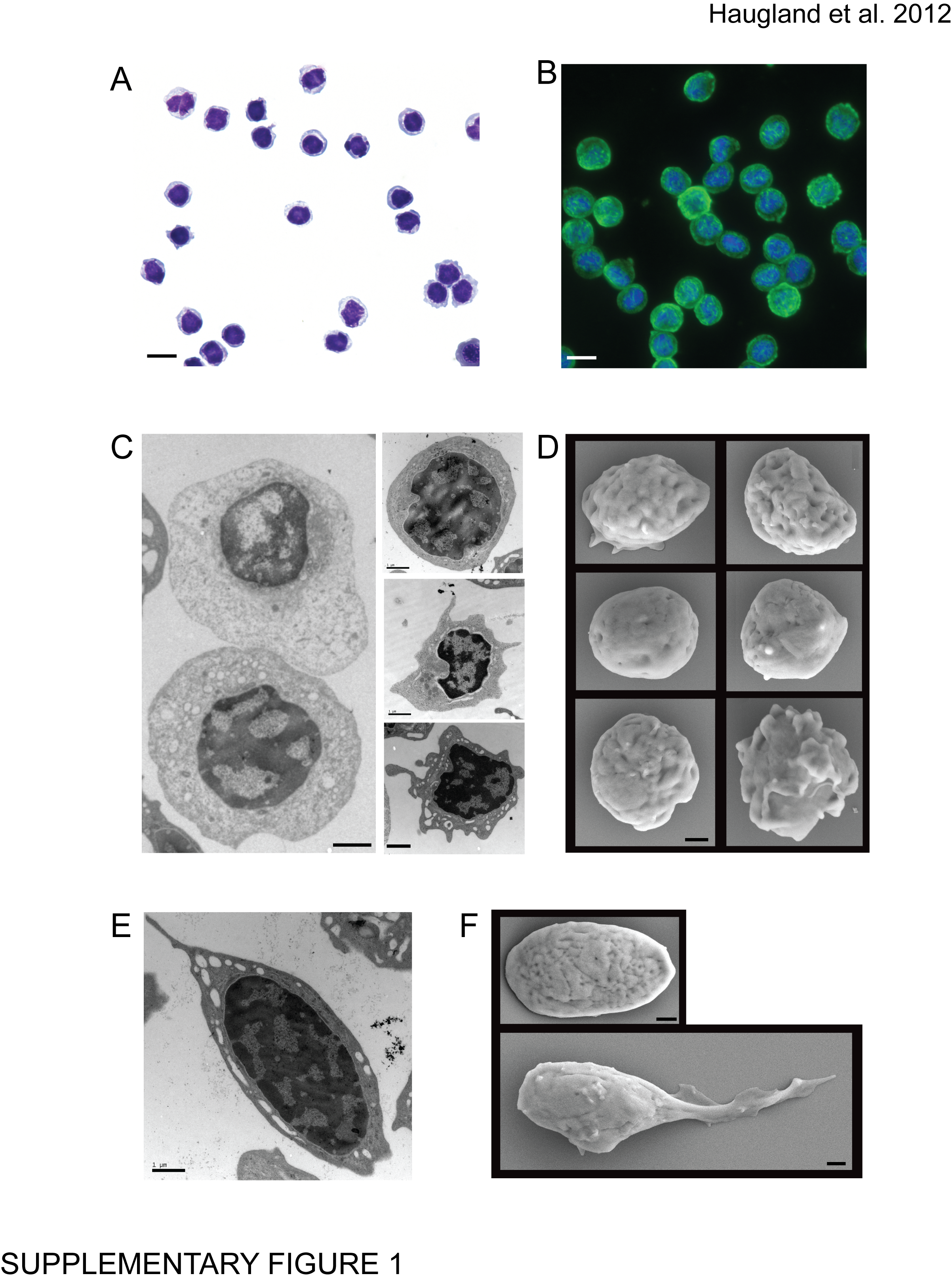

Supplement: Figure S1 — Morphological analyzes of a suspension of purified C4B6− cells using Diff Quick staining, immunostaining and electron microscopy. (A) Diff Quick staining (magnification 630×, scale bar = 5 µm). (B) Immunostaining using the polyclonal anti-TO antiserum (magnification 630×, scale bar = 5 µm). (C) TEM of representative cells (magnification left panel: 5 000×, right panels: 20 000×, scale bar = 1 µm). (D) SEM of representative cells (magnification 10 000×, scale bar = 1 µm). (E) TEM of a typical thrombocyte is shown for comparison (magnification 15 000×, scale bar = 1 µm). (F) SEM of an erythrocyte precursor, upper panel (magnification 10 000×, scale bar = 1 µm) and a typical thrombocyte, lower panel (magnification 5 000×, scale bar = 1 µm) is shown for comparison. (TIF) [file pone.0049260.s001.tif]
